# Small for gestational age births among South Indian women: temporal trend and risk factors from 1996 to 2010

**DOI:** 10.1186/s12884-015-0440-4

**Published:** 2015-02-03

**Authors:** Tunny Sebastian, Bijesh Yadav, Lakshmanan Jeyaseelan, Reeta Vijayaselvi, Ruby Jose

**Affiliations:** Department of Biostatistics, Christian Medical College, Vellore, India; Department of Obstetrics and Gynecology Unit IV, Christian Medical College, Vellore, 632002 India

**Keywords:** Small for Gestational Age, Birth weight, Risk factors, Incidence, Trend, Obstetric variables

## Abstract

**Background:**

The birth weight and gestational age at birth are two important variables that define neonatal morbidity and mortality. In developed countries, chronic maternal diseases like hypertension, diabetes mellitus, renal disease or collagen vascular disease is the most common cause of intrauterine growth restriction (IUGR). Maternal nutrition, pregnancy induced hypertension, chronic maternal infections, and other infections such as cytomegalovirus, parvovirus, rubella and malaria are the other causes of IUGR. The present study examines the secular trend of Small for Gestational Age (SGA) over 15 years and risk factors for SGA from a referral hospital in India.

**Methods:**

Data from 1996 to 2010 was obtained from the labour room register. A rotational sampling scheme was used i.e. 12 months of the year were divided into 4 quarters. Taking into consideration all deliveries that met the inclusion criteria, babies whose birth weights were less than 10^th^ percentile of the cut off values specific for gestational ages, were categorized as SGA. Only deliveries of live births that occurred between 22 and 42 weeks of pregnancy were considered in this study. Besides bivariate analyses, multivariable logistic regression analysis was done. Nagelkerke R^2^ statistics and Hosmer and Lemeshow chi-square statistics were used as goodness of fit statistics.

**Results:**

Based on the data from 36,674 deliveries, the incidence of SGA was 11.4% in 1996 and 8.4% in 2010. Women who had multiple pregnancies had the higher odds of having SGA babies, 2.8 (2.3-3.3) times. The women with hypertensive disease had 1.8 (1.5-1.9) times higher odds of having SGA. Underweight women had 1.7 (1.3 - 2.1) times and anaemic mothers had 1.29 (1.01 - 1.6) times higher odds. The mothers who had cardiac disease were 1.4 (1.01 - 2.0) times at higher odds for SGA. In teenage pregnancies, the odds of SGA was 1.3 (1.1 - 1.5) times higher than mothers in the age group 20 to 35 years.

**Conclusions:**

There is a significant reduction in the incidence of SGA by 26% over 15 years. The women with the above modifiable risk factors need to be identified early and provided with health education on optimal birth weight.

## Background

The birth weight and the gestational age at birth are two important variables which define neonatal morbidity and mortality [1]. In developing countries, the majority of low birth weight children have intra- uterine growth restriction rather than being born preterm [2].

Of 135 million children born in low income and middle income countries (LMIC) in 2010, an estimated 29.7 million were born full term but small for gestational age (SGA). A little over ten million were born preterm and appropriate for gestational age, and 2.8 million were born preterm and SGA [3]. The prevalence of SGA was 8.3% [4]. Chronic maternal vascular disease due to hypertension, diabetes mellitus, renal disease, or collagen vascular disease was the most common cause of IUGR in developed countries. Less frequently, IUGR may be due to first or second trimester foetal infection, including cytomegalovirus, malaria, parvovirus, and rubella. The majority of foetal aetiologies lead to early gestation symmetric IUGR. Hypercoagulable maternal conditions such as thrombophilia and antiphospholipid antibody syndrome also inhibit growth either by placental thrombosis formation or by secondary effects of maternal disease. Persistent maternal hypoxia due to high altitude, severe pulmonary or cardiac disease, and/or severe chronic anaemia limits oxygen delivery to the foetus and attenuates foetal growth [5-7].

In addition to short term medical morbidities that affect infants with Preterm births (PTB) and SGA, long term consequences involving neurological, cardiovascular and metabolic conditions have been found to persist into adolescence and adulthood [8,9]. These health consequences result in rising societal costs incurred through increased health care requirements and special educational needs [10].

It is known that infants born to Asian Indian mothers weigh less on average than American or European mothers. Though the Asian Indian mothers had lowest percentage of risk factors for SGA such as teenage deliveries and high parity for age, still the rate of SGA was higher amongst Asian Indian mothers. The different patterns of growth observed among Asian-Indian infants may be attributable to a different body habits and may be due genetic [11,12]. The present study examines the temporal trend of SGA over 15 years and risk factors for SGA from a referral hospital in India.

## Methods

### Ethical approval

The study was approved by the Institutional Review Board of Christian Medical College, Vellore [IRB Min. No. 7109 dated 10.03.2010].

### Study site and population

The Christian Medical College and Hospital (CMCH), Vellore in the state of Tamil Nadu, India caters to 47,110 outpatients and 15,662 inpatients per year. The Department of Obstetrics and Gynaecology on an average delivered 20 babies per day in 1996 and 40 babies per day in 2010. There were an estimated 164,250 deliveries from 1996 to 2010. Women of different socioeconomic status attend this institution for delivery including private patients of high socioeconomic status and low and middle income women who constitute the bulk of the clientele. In general women do not smoke although passive smoking from exposure to husband’s smoking or from cooking on open fire is common. Data on smoking or on method of cooking was not routinely collected. Pregnant women with HIV and STI get free treatment in the Government Hospitals around Vellore, and hence they very infrequently attend CMCH and this data has also not been included in this analysis.

### Sampling

To study the trends, distribution of different outcomes and the risk factors over the 15 year period and with limited capacity for data extraction and entry we opted for rotational sampling [13]. Briefly this method of sampling involves sequential months from the four quarters of a one year period. In 1996 data from each quarter from the months of January, April, July and October were retrieved and entered. In 1997 the respective months were February, May, August and November. This rotation of months in each quarter was continued for the 15 year period.

There was no computerization of the antenatal data prior to 2000 and the original records have been destroyed. The Labour Room Register data has been preserved and this is from where the present paper has been compiled. The antenatal assignment of GA by LMP or by US was not available from the Labour Room Register. Hence it was impossible to use LMP or US as a factor in the analysis.

### Antenatal assessment of gestational age

If menstrual cycles were regular and abdominal examination findings correlated then the last menstrual period (LMP) was taken as the best estimate of gestational age. If menstrual cycles were irregular or the LMP was unknown a dating scan at the first antenatal visit for women in first and second trimester was performed. Hadlocks formula was used for estimation of gestational age. CRL measurement was used up to 13 weeks of gestation. From 14 weeks gestation onwards, the average of the BPD, HC, FL and AC measurements were used [14]. Some patients included in the cohort were treated with in vitro fertilization and their gestation was calculated by the days since oocyte retrieval or co-incubation and adding 14 days [15].

### Dubowitz assessment of gestational age at birth

Post natal assessment of gestational by the Dubowitz assessment since 1996 the Dubowitz gestational age assessment was done on every new born admitted to the neonatal nursery for SGA or prematurity within 24 hours of birth by trained Paediatrician or Paediatric Resident. Over the period the same cadre of staff have been responsible for conducting the Dubowitz assessment. Irrespective of the method of dating antenatally the assignment of preterm or SGA was reassigned based on the Dubowitz gestational age assessment. All neonates admitted into the Nursery were directly overseen by a Professor of Neonatology at least twice a day to maintain good standard of care [16].

### Ultrasound (US)

Ultrasound for estimating foetal weight was done only when there was a suspicion of SGA clinically by symphysiofundal height measurement, or if the woman had risk factors for SGA such as previous SGA, heart disease, hypertensive disease, renal disease, collagen vascular disease. When performing ultrasound scan for suspected SGA the amniotic fluid index and the Doppler wave form of the umbilical artery were assessed. Once SGA was suspected, all these women had intensive foetal surveillance by modified Biophysical profile [17] and doppler analysis of the umbilical artery twice a week or more frequently if required. If there was foetal compromise at any time or if the gestational age was 37 weeks a planned induction or imminent delivery by caesarean section was planned.

### Inclusion and exclusion criterion

Deliveries of live births with a gestational age between 22 and 42 weeks were included irrespective of whether the women conceived spontaneously, by ovulation induction or IVF [18].

### Antenatal care procedures

All women had Haemoglobin (Hb) estimation by automated cell counter using a Coulter machine, at booking (usually in the first or early second trimester). This was repeated in the third trimester around 32 weeks. Unbooked women had Hb estimation at admission into labour room at the time of delivery. All women booked for their pregnancy had iron and calcium supplements commencing after the first trimester.

During the period 1996 to 2010, risk based screening with 100 grams; 3 hour oral Glucose tolerance test was followed. Risk factors included Asian ethnicity, increased maternal age, family history of Diabetes mellitus, obesity, multiple pregnancies, persistent glucosuria during pregnancy, repeated UTI in pregnancy [19].

### Definitions

#### Literacy

Standard of education was grouped separately for the woman and for her husband into: illiterate including those who were unable to read or write; higher secondary included those who had a school education of 12 years; and college and above for those with higher education.

#### Gestational hypertension

Gestational hypertension was grouped into hypertensive disease of pregnancy which included women who had blood pressure (B.P.) greater or equal to 140/90 mmHg on 2 occasions, 6 hours apart after 20 weeks gestation. Chronic hypertension if the diagnosis of hypertension predated the pregnancy, or had hypertension diagnosed prior to 20 weeks of gestation.

#### Cardiac disease

Cardiac Disease included all pregnant women with rheumatic or congenital heart disease diagnosed by the Cardiologist, based on echocardiogram and/or other relevant tests.

#### Body mass index (BMI)

Body mass index (BMI) was calculated from weigh at delivery using the standard formula: [weight (kg)/ height (m) ^2^] formula. Women with a BMI below18.5 kg/m^2^ were classified as underweight, normal weight for BMI of 18.5 – 24.9 kg/m^2^, overweight 25–29.9 kg/m^2^ and obese for BMI > = 30 kg/m^2^ [20].

#### Anaemia

Anaemic women were those with a Haemoglobin (Hb) <11 g/dL based on World health Organization (WHO) definition of anaemia [21].

#### Diabetes

Diabetes included women with diabetes predating their pregnancy and those with a positive 100 gm 3 hour oral glucose tolerance test with cut-offs at baseline, one, two and three hours following glucose ingestion.

#### Oligohydramnios

Oligohydramnios was an amniotic fluid index of <5 cms.

#### Birth weight

All babies were weighed within an hour of delivery using 111 Braun electronic weighing scale. The scale is calibrated regularly by the Engineering department, so as to maintain accuracy of the scale. The accuracy of the scale is ± 0.5 g.

#### Pre and post term

Delivery at a gestational age of less than 37 weeks was classified as preterm and after 42 weeks as post term by the gestational age established from the Dubowitz assessment [16].

#### Small for gestational age (SGA)

SGA was assigned when a new born had a birth weight lower than the 10^th^ centile for gestational age week.

#### Appropriate for gestational age (AGA)

AGA was assigned when a new born had a birth weight between 10th and 90th centile for gestational age week.

### Statistical methods

The association between risk variables and SGA were tested using Chi-square test with Yates correction. The variables which were significant at *P* < 0.25, at Bivariate analysis were considered for stepwise logistic regression analysis. Nagelkerke R^2^ statistics and Hosmer and Lemeshow chi- square statistics were used as goodness of fit statistics. P value < = 0.05 was considered for statistically significance. The results were presented with OR and 95% CI. SPSS 16.0 was used to analyze data.

## Results

**Figure 1:**
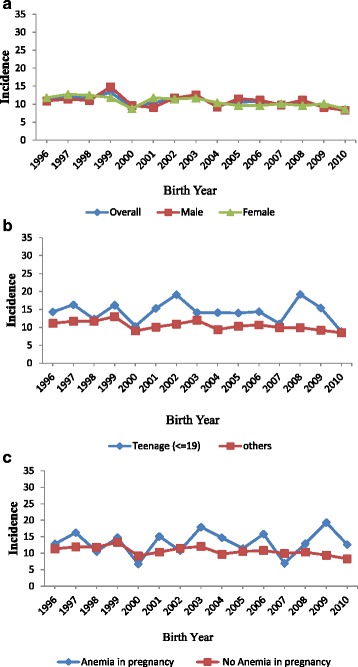
**Incidence of Small for Gestational Age (SGA) by infant’s gender, maternal age and maternal anaemia from 1996 to 2010. a**: Incidence of Small for Gestational Age (SGA) by overall and by gender of infant. **b**: Incidence of Small for Gestational Age (SGA) based on maternal age groups. **c**: Incidence of Small for Gestational Age (SGA) by maternal anaemia status.

**Figure 2:**
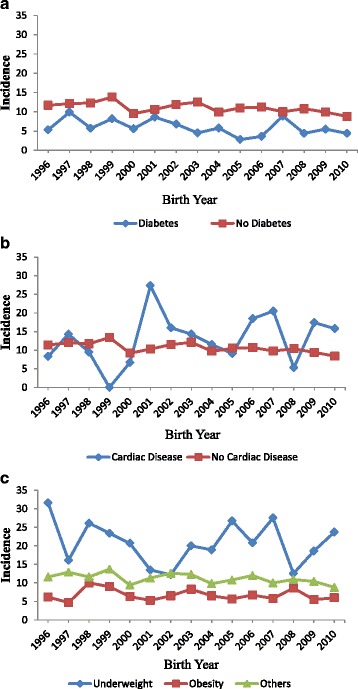
**Incidence of Small for Gestational Age (SGA) by diabetes, cardiac disease and BMI at delivery from 1996 to 2010. a**: Incidence of Small for Gestational Age (SGA) in mothers by diabetes status. **b**: Incidence of Small for Gestational Age (SGA) in mothers by cardiac disease status. **c**: Incidence of Small for Gestational Age (SGA) in mothers by BMI categories.

In total, there were 41,055 live births. Of these, 36,674 deliveries were registered through the outpatient clinics and 3867 SGA babies were observed during this 15 year period in this group. The mean (sd, min and max) of the mothers was 25.2 (4.2, 14 and 49) years. Of these mothers 5.9% were teenage mothers. The mean (sd, min and max) height, weight and BMI was 155.4 cm (6.1, 120 and 189); 62.9 kg (11.4, 30, 140) and 26 (4.3, 9.0, 57.4) respectively. While women with anaemia constituted 2%, the proportion of women with severe anaemia was very low. The prevalence of hypertension and diabetes was 7.8% and 6.6% respectively. Of these deliveries 13.1% was preterm deliveries. The mean (SD), median (IQR) of birth weight and gestational weeks were 2.9 kg (0.59), 2.9 (2.6, 3.2) kg and 38 (2.4), 39 (38, 40) respectively. The incidence of SGA was about 11.5% (11% - 12%) from 1996 until 2003 and reduced to 9.8% (9.4% - 10.2%) from 2004. A declining trend in the prevalence of SGA from 2004 was thus observed. There were 18,795 (51%) male babies and 17,810 (49%) female babies in the sample. For 70 babies information about their gender was missing. The incidence of SGA in each year for male and female babies with different risk variables is presented in Table 1. The results show no significant difference in the incidence of SGA between male and female babies. Of the deliveries, 13.1%, 86.2% and 0.7% were pre term, full-term and post term deliveries respectively. The diagrammatic displays of the incidence of SGA based on the presence of risk factors are presented in Figures 1 and 2.

**Table 1:** **Incidence of Small for Gestational Age from year 1996 – 2010 according to gender and risk factors**

**Birth year**	**Small for gestational age**																
	**Total delivery studied**	**Overall**		**Male**		**Female**		**Teenage (<=19)**		**Cardiac disease**		**Anaemia in pregnancy**		**Obesity**		**Diabetes**	
		**N**	**%**	**n**	**%**	**n**	**%**	**n**	**%**	**n**	**%**	**n**	**%**	**n**	**%**	**n**	**%**
1996	1661	189	11.4	91	10.9	98	11.8	16	14.3	1	8.3	10	12.8	8	6.2	4	5.3
1997	1745	209	12.0	102	11.4	107	12.7	21	16.3	2	14.3	6	16.2	8	4.7	9	9.9
1998	1835	215	11.7	106	11.0	109	12.5	14	12.3	2	9.5	6	10.5	17	10.0	9	5.7
1999	2036	271	13.3	154	14.8	117	11.8	22	16.2	0	0.0	10	14.7	15	9.0	14	8.2
2000	1934	177	9.2	99	9.6	78	8.7	16	10.3	1	6.7	1	6.7	14	6.3	11	5.6
2001	1868	195	10.4	85	9.1	110	11.8	19	15.3	3	27.3	8	15.1	15	5.3	13	8.6
2002	2408	277	11.5	146	11.7	131	11.4	31	19.1	4	16.0	4	10.8	22	6.5	14	6.8
2003	2673	325	12.2	177	12.6	148	11.7	30	14.1	2	14.3	10	17.9	27	8.3	5	4.5
2004	2745	268	9.8	130	9.2	138	10.4	27	14.1	3	11.5	5	14.7	27	6.5	7	5.8
2005	2366	249	10.5	136	11.5	113	9.6	22	14.0	2	9.1	4	11.4	21	5.7	4	2.8
2006	2521	273	10.8	141	11.1	132	9.6	18	14.4	5	18.5	3	15.8	34	6.7	4	3.6
2007	2909	288	9.9	143	9.8	145	10.1	15	11.0	8	20.5	2	6.9	32	5.8	14	8.9
2008	2830	292	10.3	155	11.1	137	9.6	24	19.2	1	5.3	4	12.9	49	8.6	9	4.4
2009	3260	311	9.5	155	9.1	156	10.1	23	15.4	8	17.4	11	19.3	36	5.5	13	5.5
2010	3883	328	8.4	166	8.3	162	8.6	12	8.8	3	15.8	15	12.6	48	6.0	13	4.4
Total	36674	3867		1986		1881		310		45		99		373		143

### Demographic risk factors

The distribution of risk variables for SGA and the results of multivariable analysis are presented in Table 2. Teenaged mothers (< =19 years) were 1.31 (1.12 -1.54) times at higher odds for having SGA babies as compared to mothers who were between 25–29 years of age (p < 0.001). Women whose husbands worked as unskilled labourers were 1.10 (1.01-1.22) times at higher odds for SGA as compared to those whose husbands were professionals (p < 0.04). Women whose husbands were involved in agricultural work or were businessmen had 0.85 (0.75 - 0.95) times significantly lower odds for SGA compared to those whose husbands were professional (p < 0.005). Further, women whose husbands were illiterate (OR = 1.39, 1.11 – 1.73) and those whose husbands had only completed 12 years of schooling were (OR = 1.13, 1.01 – 1.26) also at higher odds for having SGA babies as compared to those women whose husbands had completed graduation and above. In addition, women who were illiterate or who had only completed 12 years of schooling were 1.41 (1.12 - 1.77) and 1.25 (1.11 – 1.40) times at higher odds for having SGA babies respectively as compared to women who had completed graduation and above (p < 0.001).

**Table 2:** **Risk factors for small for gestational age based on bivariate (unadjusted) and multivariate (adjusted) analyses**

**Risk factor variables**	**Unadjusted analysis (N = 36674)**					**Adjusted analysis**		
	**SGA**				**p value**			
	**No**		**Yes**			**OR**	**95% CI**	**p value**
	**n**	**%**	**n**	**%**				
Gender:								
Male	16809	89.4	1986	10.6	1.00			
Female	15928	89.4	1882	10.6				
Age in years:								
25–29	12427	90.8	1252	9.2	<0.001	1.00		
<=19	1855	85.7	310	14.3		1.31	1.12 – 1.54	0.001
20–24	13187	88.4	1733	11.6		1.09	0.99 – 1.19	0.07
30–34	4232	90.4	447	9.6		1.12	0.99 – 1.28	0.08
> = 35	990	89.9	111	10.1		1.11	0.86– 1.42	0.43
Husband Occupation:								
Professional	15786	90.7	1622	9.3	<0.001	1.00		
Agriculture/Business	5681	91.3	541	8.7		0.85	0.75 – 0.95	0.005
Unskilled/Others	10419	86.8	1583	13.2		1.11	1.01 – 1.22	0.04
Husband Education:								
Degree and Above	12550	92.2	1069	7.8	<0.001	1.00		
Higher Secondary	16314	88.2	2189	11.8		1.13	1.01 – 1.26	0.03
Illiterate	1224	83.9	235	16.1		1.39	1.11 – 1.73	0.004
Mother Occupation:								
Professional	2765	92.9	211	7.1	<0.001			
Housewife	29352	89.1	3578	10.9				
Unskilled/Others	272	90.4	29	9.6				
Mother Education:								
Degree and Above	10849	92.5	882	7.5	<0.001	1.00		
Higher Secondary	18249	88.2	2432	11.8		1.25	1.11 – 1.40	<0.001
Illiterate	1200	84.6	218	15.4		1.41	1.12 – 1.77	0.003
Hypertension:								
No	29376	90.0	3265	10.0	<0.001	1.00		
Yes	3431	85.1	603	14.9		1.77	1.58 – 1.98	<0.001
Multiple Pregnancy:								
No	31868	89.9	3593	10.1	<0.001	1.00		
Yes	939	77.3	275	22.7		2.77	2.35 – 3.28	<0.001
Cardiac disease:								
No	32521	89.5	3823	10.5	0.07	1.00		
Yes	286	86.4	45	13.6		1.42	1.01 – 1.99	0.04
Oligohydramnios:								
No	32695	89.5	3822	10.5	<0.001	1.00		
Yes	112	70.9	46	29.1		3.67	2.49– 5.40	<0.001
Anaemia in pregnancy:								
No	32181	89.5	3769	10.5	0.006	1.00		
Yes	626	86.3	99	13.7		1.29	1.01 – 1.65	0.04
Miscarriage:								
No	26972	89.1	3296	10.9	<0.001	1.00		
Yes	5835	91.1	572	8.9		0.83	0.74 – 0.92	0.001
BMI:								
Normal weight	11835	86.7	1808	13.3	<0.001	1.00		
Underweight	506	79.1	134	20.9		1.71	1.38 – 2.13	<0.001
Overweight	11192	91.3	1063	8.7		0.64	0.58 – 0.70	<0.001
Obesity	5301	93.4	373	6.6		0.46	0.40 – 0.52	<0.001

### Obstetric rick factors

The odds of SGA in babies was 1.77 (1.58 – 1.98) times higher for women whose pregnancies were complicated by hypertensive disorders as compared with those who did not have hypertension (p < 0.001). The finding of Oligohydramnios was higher when the neonate was SGA by 3.67 (2.49-5.4) times as compared with women whose babies were appropriate for gestational age (p <0.001).

In women who had multiple pregnancies the odds of having an SGA neonate was 2.77 (2.35 – 3.28) times higher than women who had only a single pregnancy (p < 0.001). Similarly, women who had cardiac disease were 1.42 (1.01 – 1.99) times at higher odds for SGA babies as compared to women who did not have cardiac disease (p = 0.04). The odds of having an SGA neonate was 1.29 (1.01 – 1.65) times higher for women who were anaemic during their pregnancy compared to those who were not anaemic (p = 0.04). Underweight women were 1.71 (1.38 – 2.13) times at higher odds for having SGA babies as compared to those women who were of normal weight (p < 0.001). Lastly, overweight 0.64 (0.58 - 0.70) and obese women had 0.46 (0.74 - 0.92) times lower odds for SGA as compared to women with normal weight (p < 0.001). Women with a previous history of miscarriage had 0.83 (0.74 - 0.92) times significantly lower odds for having SGA babies (p < 0.01).

## Discussion

Birth weight has been used as a strong predictor of neonatal, infant and child mortality. In the absence of ultrasound estimation of foetal weight, birth weight at each gestational age is used as a proxy for actual foetal growth [22]. This study attempted to find the incidence of the trend for SGA in this population and the risk factors associated with having SGA babies.

This is a large scale study from a referral hospital in India, which caters predominantly to obstetric care of the local population besides referrals. The women do not smoke (though passive smoking from exposure to husband’s smoking or from cooking on open fire is common). There is a mixture in the socioeconomic status of women who come for delivery at this institution. There is a mix of private patients, (who belong to the high socioeconomic status) and low and middle income women who constitute the bulk of the clientele. Results from our study showed that the rate of SGA babies had declined significantly from 11.4% in 1996 to 8.4% in 2010, that is, 26.3% reduction in 15 years (p < 0.001). We defined SGA as less than the 10^th^ percentile birth weight value from the same cohort of children delivered at this hospital, and specific to both male and female gender rather than base it on the Canadian foetal growth standard or the old Indian standard [17]. Kumar *et al*. [13] have provided birth weight standards for various gestational ages from the same cohort of children from the same department. The incidence of SGA in our study was 11.5% or 115 per 1000 births and the trend of SGA has been declining over these years. The overall stillbirth rate during the last fifteen years was 26 per 1000 births. As in the SGA rate, the trend of stillbirth rate has been declining over these years. However, of the SGA, the contribution due to stillbirth is 22.6%. Therefore, stillbirths might have small impact in the SGA rate. Lee *et al*. [23] have reported that the incidence of SGA for Asian Indian infants was 14.5% while this was 2.7% for Caucasian, where the SGA classification was based on birth weight less than the 10^th^ percentile for gestational age. Ananth *et al.* [24] have reported that the incidence rates of SGA in the white population was 9.8% in 1989 and 9% in 1998 and this was 19.4% and 17.4% in the black population in 1989 and 1998 respectively. Madan *et al.* [25] have reported that foreign born Indians in the United States had a 6.3% SGA. No Indian standard has been established in the recent past [17].

George *et al.* [26] have reported high rates of SGA when the foetal growth was considered against the Canadian foetal growth standard. We are skeptical in using Indian standards which were reported in 1996 [17] in view of the socio demographic transition that has happened over the last 40 years in terms of improved access to care, good nutrition etc. [27]. A publication from the same department in 1996 did not provide actual percentiles and dealt with restricted gestational age from 29 to 42 weeks [17]. However, the current study had a range of gestational age from 22 to 42 weeks. However, all the above studies have used the US based reference standard to classify SGA [28]. Our data has shown a similar rate as compared to the above study. They have also reported that rates of chronic hypertension, pregnancy induced hypertension that are risk factors for SGA have increased more in blacks than in the Whites in United States.

When taking into consideration the demographic risk factors for SGA, this study found that women, who were illiterate, were at higher risk for having SGA babies. Husbands of these women also reflected the same literacy and unskilled work characteristics. This could be explained by the fact that awareness of good nutrition and rest was absent or not available to this group of women and husbands. Their economic condition would not have been conducive to good nutrition as well. Their financial status, we surmise, precluded adequate antenatal care, nutrition and support from family as well.

Teenage pregnancies showed a higher risk for SGA, according to this present study. This could reflect the lack of awareness of antenatal care, physiologic inability to have an appropriate for gestational age neonate or even the increased prevalence of hypertensive disorders which was found more often in this sub group.

Women with obstetric risk factors such as teenage pregnancy, hypertensive disorders, multiple pregnancy, cardiac disease, anaemia in pregnancy and underweight, were significantly at higher risk for having SGA babies. Mavalankar *et al.* [29] reported that anaemia, primiparity, poor obstetric history, lack of antenatal care and hypertension were significant risk factors for SGA. Oligohydramnios was found to be significantly associated with SGA.

Our study has shown a 26% reduction in SGA over the last 15 years, probably due to reductions of some of the risk factors in the community. The percentage of teenage pregnancies decreased from 7.1% prior to 2004 to 4.7% after 2004 and the reduction was statistically significant (Table 3). The percentage of overweight and obese women have significantly increased after 2004, suggesting that there has been an improvement in the nutritional status of women (from underweight) and therefore a decline in SGA. Similarly the education level of women has increased (degree and above: 29% vs 40%), which probably contributed to a reduction in SGA as well. There are very few treated/ untreated malarial pregnant women among the women delivering in CMCH. This area is not endemic for malaria. Pregnant women with HIV and STI get free treatment in the Government Hospitals around Vellore, and hence very infrequent in this Hospital.

**Table 3:** **Distributions of risk variables before and after 2004**

**Risk variables**	**<=2004**		**>2004**		**p value**
	**N**	**%**	**n**	**%**	
Age:					
<=19	1337	7.1	828	4.7	0.03
>19	17507	92.9	16871	95.3	
Hypertension:					
No	16967	89.75	15673	88.20	0.12
Yes	1938	10.25	2096	11.80	
Multiple Pregnancy:					
No	18273	96.66	17187	96.72	0.95
Yes	632	3.34	582	3.28	
Cardiac disease:					
No	18746	99.16	17597	99.00	0.88
Yes	159	0.84	172	1.00	
Anaemia in pregnancy:					
No	18470	97.70	17479	98.37	0.53
Yes	435	2.30	290	1.63	
Miscarriage:					
No	15609	82.56	14659	82.50	0.95
Yes	3296	17.44	3110	17.50	
BMI:					
Underweight	425	2.51	215	1.41	0.33
Normal weight	8153	48.04	5489	36.02	<0.001
Overweight	6175	36.38	6080	39.90	0.001
Obesity	2219	13.07	3455	22.67	<0.001
Mother Occupation:					
Professional	1293	6.89	1683	9.65	0.01
Housewife	17377	92.55	15552	89.22	<0.001
Mother Education:					
Degree and Above	4788	28.96	6943	40.14	<0.001
Higher Secondary	10927	66.09	9753	56.40	<0.001

Not all women had a dating scan in the first trimester. When menstrual cycles were regular, and findings at examination were corresponding, LMP was used for gestational age. If menstrual cycles were irregular, dating by scan was used, at the first visit (first or second trimester. In case of in vitro fertilisation, calculating days since oocyte retrieval or co-incubation and adding 14 days [15]. The being a retrospective study, the exact numbers of pregnant women who had a dating scan, could not be ascertained.

Some of the risk factors for SGA are modifiable, and care should be taken to correct these, so that SGA can be further decreased. Among the demographic risk factors, teenaged pregnancies can be avoided with education and awareness of the parents and school going female students. Literacy levels are related to prevalence of SGA; the higher the literacy levels, the lower the SGA prevalence. One of the modifiable obstetric risk factors are underweight mothers, and this can be brought down with education and awareness of nutrition among couples. A major modifiable obstetric risk factor is the presence of anaemia in pregnant women. This constitutes one of the causes for increased maternal mortality and morbidity, in addition to SGA babies as well. Mothers with multiple pregnancies and hypertensive disorders of pregnancy should have special antenatal care to avert mortality and morbidity in their SGA babies and themselves as well.

This study has several limitations including that it is impossible to get the proportion of SGA calculated by ultrasound measurement and by dates (LMP) separately, since there was no computerisation of the antenatal data prior to 2000. The Labour Room Register data was preserved, from where the present paper has been compiled. Though this is a large scale study representing 15 years of deliveries from a private referral teaching hospital, this may not represent the deliveries which are taking place at the Primary Health Centres in the rural area. Some of the changes in the secular trend could be attributed to the policy changes in the hospital such as an increase in staff-patient ratio, increase in the number of units in the department, dedicated women and children blocks and the availability of consultants at any time point. Overall decrease in the incidence of SGA could also be attributed to the availability of high technology instruments which have been made available over time and thus helped in diagnosing and treating the SGA earlier. Moreover, gold standard ultrasound was not available to all women in this cohort which is a situation repeatedly observed in many low income countries. In addition the records for the women for whom it was available could not be retrieved. Nevertheless the large population of women evaluated here with consistent dating using the same method of Dubowitz gestational age assessment still permitted an evaluation of the SGA trends across time. However, we do not have such data to correlate these changes with the incidence of SGA.

## Conclusion

The rates of SGA were 11.4% and 8.4% in 1996 and 2010 respectively. There is a significant reduction in the incidence of SGA by 26%. The number of teenaged childbirths had decreased significantly. The literacy levels were significantly higher probably resulting in better awareness and lesser SGA babies. The number of women with low Body Mass Index decreased and so SGA had come down as well. Pregnancy with SGA neonate was significantly associated with oligohydramnios. Teenage pregnancy, hypertensive disorders, multiple pregnancies, cardiac disease, anaemia in pregnancy and being underweight during pregnancy were significant risk factors for having SGA babies. Therefore the women who seek antenatal care with the above risk factors need to be identified early and health education regarding safe delivery and optimal birth weight has to be imparted periodically.

## Consent

The Institutional Review Board of Christian Medical College Vellore provided consent for this research paper as recorded in the following minutes (IRB Min. No. 7109 dated 10.03.2010).
